# Impact of triglycerides and waist circumference on insulin resistance and β-cell function in non-diabetic first-degree relatives of type 2 diabetes

**DOI:** 10.1186/s12902-021-00788-5

**Published:** 2021-06-16

**Authors:** Fahd Ahmed, Molham AL-Habori, Ebtesam Al-Zabedi, Riyadh Saif-Ali

**Affiliations:** Department of Biochemistry and Molecular Biology, Faculty of Medicine and Health Sciences, University of Sana’a, Sana’a, Republic of Yemen

**Keywords:** First-degree relatives of Type 2 DM, Insulin resistance, β-cell function, Metabolic syndrome, HbA1c, Fructosamine

## Abstract

**Background:**

Although there is abundant evidence indicating the relative contribution of insulin resistance (HOMA-IR) and β-cell dysfunction (HOMA-β) among first-degree relatives (FDRs) of Type 2 DM patients, few studies reported the association between HOMA-IR and HOMA-β with metabolic syndrome. Our objective was to evaluate the impact of metabolic syndrome factors on HOMA-IR, HOMA-β and glycoproteins in non-diabetic FDRs.

**Methods:**

In this study, 103 Yemeni male subjects aged 25–42 years, with BMI < 25 kg/m^2^ were examined, 39 of whom were normal subjects with no family history of diabetes served as control and 64 subjects were non-diabetic FDRs of Type 2 DM patients.

**Results:**

Both glycoproteins, glycated haemoglobin (HbA1c) and fructosamine as well as insulin, HOMA-IR and HOMA-β were significantly (*p* = 4.9 × 10^−9^; 6.0 × 10^−8^; 6.6 × 10^−12^; 1.3 × 10^−7^; 5.5 × 10^−12^, respectively) higher in non-diabetic FDRs as compared to control group. Fasting plasma glucose, though within normal range, were significantly (*p* = 0.026) higher in non-diabetic FDRs. Linear regression analysis showed that both TG and WC are the main metabolic syndrome factors that significantly increased HOMA-IR (B = 0.334, *p* = 1.97 × 10^−6^; B = 0.024, *p* = 1.05 × 10^−5^), HOMA-β (B = 16.8, *p* = 6.8 × 10^−5^; B = 0.95, *p* = 0.004), insulin (B = 16.5, *p* = 1.2 × 10^−6^; B = 1.19, *p* = 8.3 × 10^−6^) and HbA1c (B = 0.001, *p* = 0.034; B = 0.007, *p* = 0.037).

**Conclusion:**

Triglyceride and WC are the important metabolic syndrome factors associated with insulin resistance, basal β-cell function and insulin levels in non-diabetic FDR men of Type 2 DM patients. Moreover, FDRs showed insulin resistance with compensatory β-cell function (hyperinsulinaemia) suggesting that insulin resistance precede the development of pancreatic β-cell dysfunction in individuals at risk of Type 2 DM.

## Background

Type 2 Diabetes Mellitus (DM) is the predominant form of diabetes and accounts for approximately 90% of all diagnosed cases. A recent estimate by the International Diabetes Federation reported the global diabetes prevalence to be 9.3% (463 million) in 2019, rising to 10.9% (700 million) by 2045 [1]. Type 2 DM is a heterogeneous group of disorders that display relative insulin deficiency and is usually associated with obesity, insulin resistance, impaired insulin secretion, and increased hepatic glucose production [2]. Both genetic susceptibility and environmental factors likely contribute to the development of Type 2 DM [3]. In insulin-resistant states, pancreatic islets usually respond by increasing insulin secretion to maintain normoglycaemia, a process termed β-cell compensation. Individuals that are unable to sustain the β-cell compensatory response develops Type 2 DM. Longitudinal studies of individuals that developed Type 2 DM showed a rise in insulin levels in the normoglycaemic and pre-diabetic phases that maintain glycaemia near normal despite the presence of insulin resistance (β-cell compensation), followed by a decline in insulin levels (β-cell dysfunction) when fasting glycaemia exceeds the upper limit of normal [4].

The specific etiologies of Type 2 DM are not known; however, the disease is associated with family history of diabetes, impaired glucose metabolism, obesity, physical inactivity, and ethnicity [3]. A family history of diabetes confers an up to threefold increased risk for first-degree relatives (FDRs) to develop diabetes [5]. The risk of developing Type 2 DM is 40% for individuals who have one parent with Type 2 DM and about 70% if both parents are affected [6]. A family history of diabetes has been shown to have a higher positive predictive value for diabetes than obesity [7]. Unlike diabetic patients, FDRs of diabetic patients do not display with signs of symptoms and their risk factors are often overlooked. Typical metabolic syndrome alterations have been reported at an early age, including central obesity, dyslipidemia, glucose intolerance, and high blood pressure [8, 9].

In FDRs, both insulin resistance and pancreatic β-cell dysfunction are inseparable phenomena and have been proposed to synergistically exacerbate type 2 diabetes and increase cardiometabolic risk [2, 10, 11]. During the past three decades, the relative contribution of insulin resistance and impaired β-cell function to Type 2 DM development has been the subject of many debates. Several prospective studies involving FDRs of Type 2 DM in different ethnic groups have yielded different conclusions. Insulin resistance was reported to be an important risk factor for development of diabetes in Pima Indians [12], African-Americans [13], and Indians [14, 15]; while impaired β-cell function was suggested to have a more pronounced contribution to Type 2 DM than insulin resistance in Caucasians [16, 17], Japan [18], Korea [19], and China [20, 21]. Meanwhile, others demonstrated both insulin resistance and impaired β-cell function had occurred in FDRs of type 2 DM patients before glucose tolerance was abnormal [22].

Although there is abundant evidence indicating the relative contribution of insulin resistance and β-cell dysfunction among FDRs of Type 2 DM patients, few studies reported the association between insulin resistance and β-cell dysfunction with metabolic syndrome. Therefore, the aim of this study was to evaluate the impact of metabolic syndrome risk factors on insulin resistance, β-cell function and glycated proteins.

## Methods

### Study design, subjects and data collection

This cross-sectional study was performed on 103 Yemeni male subjects aged 25–42 years, 64 were healthy non-diabetic FDRs of subjects with Type 2 DM patients who accompanied their diabetic patients during their routine visits to the Endocrine and Diabetic Clinic of Al-Thwara Hospital, Sana’a, and 39 were healthy control subjects with no family history of diabetes, who were on no medication that may affect blood glucose or lipid profile. A family history of diabetes was considered as the presence of diabetes in parents and/or siblings. All participants enrolled in this study were male to exclude the variation in insulin sensitivity during the menstrual cycle (whereby insulin sensitivity begins to decrease near ovulation and peaks during the luteal phase in premenopausal women without diabetes; and that estradiol and progesterone levels positively associated with measures of insulin sensitivity); as well as those with body mass index (BMI) >25 kg/m^2^ in order to avoid the influence of obesity on insulin resistance. Other exclusion criteria included those with diabetes, cardiac, renal, thyroid, liver diseases (which affects albumin metabolism and plasma glucose level), blood disorders (hemolytic anemia, iron deficiency anemia, anemia of chronic diseases and others) (which leads to incorrect glycated haemoglobin, HbA_1C_), and those with infections as well as smokers (which induces various cytokines and inflammatory mediators that affect insulin resistance). The study protocol was approved by the institutional review board (IRB) of the Faculty of Medicine and Health Sciences, Sana’a University. Informed consent was obtained from all participants after explaining the purpose and nature of the study.

Standard physical examinations including clinical variables were assessed as previously described [23]. The subject’s height and weight were measured and body mass index (BMI), defined as weight in kilograms (kg) divided by height in meters squared (m^2^), was calculated. Waist circumference (WC) was measured midway between the lower rib margin and the superior iliac spine at the end of gentle expiration in a standing position. Blood pressure (BP) measurements were taken from each subject’s right arm in the seated position by using an Omron IntelliSense automatic blood pressure monitor after 10 min of rest in a quiet room. Two to three successive BP readings were obtained at 5 min intervals and averaged. Normal glucose tolerance (NGT) was confirmed by fasting blood glucose (FBG) and HbA1c. Metabolic syndrome was classified according to the IDF criteria [24], defined by the presence of central obesity (WC in Asian male ≥90 cm) together with two or more clinical features including raised triglyceride (TG), low HDL-cholesterol (HDL-c), high systolic blood pressure (SBP) and/or diastolic blood pressure (DBP), and raised FBG.

### Blood collection and biochemical analysis

The following analysis was carried out as previously described [23]. A fasting venous blood (5 ml) was collected from each subject after an overnight fast of more than 10 h and divided into two vacuum tubes; 4 ml into plain tubes for biochemical assay and 1 ml into K_2_EDTA tube for HbA1c determination. The serum was separated within 30 min and stored at −20 °C for biochemical analysis. Haemolysate was prepared immediately for HbA1c determination within 2 h of blood collection. Serum TG, HDL-c, FBG and HbA1c were measured by chemistry autoanalyzer (Siemens Healthcare Diagnostics Inc., USA) using their respective kits. Fructosamine was measured spectrophotometrically (BioSystems, Spain). Insulin was measured by electrochemiluminescence immunoassay (ECL) on the Elecsys autoanalyzer (Roche Diagnostics, Germany). Insulin resistance (HOMA-IR) and β-cell function (HOMA-β) were calculated using the homeostasis model assessment (HOMA 2) calculator v2.2 which is available from Oxford Centre for Diabetes, Endocrinology and Metabolism.

### Statistical analysis

The statistical analyses were performed on Statistical Package for the Social Sciences (SPSS) version 11.5 (SPSS Inc., Chicago, IL, USA). The sample size was calculated by “Real Statistics Resource Pack” added to excel. A medium effect size (0.35), power (0.8), 5 predicator (metabolic syndrome factors), and 0.05 significant levels was used. Data were log transformed because they were not normally distributed. These parameters means and 95% confidence intervals were transformed back and reported as geometric means. The t-test was used for comparing the diabetic and metabolic syndrome parameter between normal subjects and FDRs of Type 2 DM. The impact of metabolic syndrome factors on diabetic parameters among FDRs of Type 2 DM was assisted by multiple linear-regression (enter method) controlled for age. The correlation was done between all parameters to screen the cofactor parameters to be included in multiple linear regression analysis. Significant differences were indicated if *p*-value was <0.05.

## Results

Table 1 shows the baseline characteristics of the non-diabetic FDRs and control group. Body mass index, WC, SBP and DBP were significantly (*p* = 0.0004, *p* = 2.4 × 10^−5^, *p* = 0.038, *p* = 0.009) higher in non-diabetic FDRs by 8, 9.3, 4.5 and 5.5%, respectively as compared to control group. Plasma TG was significantly (*p* = 8.6 × 10^−7^) higher in non-diabetic FDRs than that of the control group by 51.2%; whereas plasma HDL-c was border-line significantly (*p* = 0.089) lower in non-diabetic FDRs by 6.1%. Along the same line, FBG, HbA1c and fructosamine were significantly (*p* = 0.026; *p* = 4.9 × 10^−9^, *p* = 6.0 × 10^−8^) higher in non-diabetic FDRs by 4, 6 and 19.4%, respectively with respect to control group. Plasma insulin levels, HOMA-IR and HOMA-β were significantly (*p* = 6.6 × 10^−12^, *p* = 5.5 × 10^−12^, *p* = 1.3 × 10^−7^) higher in non-diabetic FDRs by 87.3, 87.5, 37.7% as compared with the control group.

**Table 1:** Baseline characteristics of normal subjects and non-diabetic first degree relatives of Type 2 DM patients

	Normal subjects (*n* = 39)	FDRs of Type 2 DM (*n* = 64)	*p*-value
Age (years)	31.4 ± 4.61	32.3 ± 5.16	0.373
Body mass index (kg/m^2^)	21.2 ± 2.52	22.9 ± 2.11	**0.0004**
Waist Circumference (cm)	80.9 ± 7.94	88.4 ± 9.2	**2.4 × 10**^**−5**^
Systolic blood pressure (mmHg)	112 ± 11	117 ± 11	**0.038**
Diastolic blood pressure (mmHg)	73 ± 8	77 ± 8	**0.009**
Triglycerides (mmol/l)	1.31 ± 0.53	1.98 ± 0.76	**8.6 × 10**^**−7**^
HDL-cholesterol (mmol/l)	1.15 ± 0.22	1.08 ± 0.19	0.089
Fasting blood glucose (mmol/l)	4.99 ± 0.34	5.19 ± 0.47	**0.026**
Glycated hemoglobin (%)	5.0 ± 0.20	5.3 ± 0.28	**4.9 × 10**^**−9**^
Fructosamine (mmol/l)	248 ± 39.0	296 ± 41.6	**6.0 × 10**^**−8**^
Insulin (pmol/l)	38 ± 13.70	71.1 ± 28.60	**6.6 × 10**^**−12**^
HOMA-IR	0.8 ± 0.26	1.5 ± 0.60	**5.5 × 10**^**−12**^
HOMA-β	85 ± 24	117 ± 33	**1.3 × 10**^**−7**^

Data are presented as means ± SD. Mean significant difference *p* < 0.05 bolded

*HOMA-IR* insulin resistance, *HOMA-β* β-cell function

The correlation between metabolic syndrome factors and diabetic parameters is depicted in Table 2. Triglyceride and WC showed positive correlation with HbA1c, insulin, HOMA-IR and HOMA-β. Moreover, WC was positively correlated with FBG. Diastolic blood pressure, however, was positively correlated with insulin, HOMA-IR and border-line associated with HOMA-β. Unlike HbA1c, fructosamine levels did not show a correlation with any of the metabolic syndrome factors. Therefore, fructosamine, HDL-c and SBP were not included in the following linear regression analysis.

**Table 2:** Correlation of metabolic syndrome factors with diabetic parameters among FDRs of Type 2 DM

	FBG	HbA1c	Fructosamine	Insulin	HOMA-IR	HOMA-β
Age	0.15 (0.12)	**0.25 (0.01)**	0.14 (0.16)	−0.05 (0.60)	−0.05 (0.61)	−0.13 (0.19)
Waist Circumference	**0.26 (0.009)**	**0.27 (0.007)**	0.11 (0.25)	**0.50 (6.5 × 10**^**−8**^**)**	**0.50 (8.6 × 10**^**−8**^**)**	**0.38 (9 × 10**^**−5**^**)**
Triglyceride	0.1 (0.60)	**0.2 (0.02)**	0.2 (0.07)	**0.5 (4.7 × 10**^**−9**^**)**	**0.5 (7.4 × 10**^**−9**^**)**	**0.5 (3.2 × 10**^**−7**^**)**
Systolic Blood Pressure	0.11 (0.27)	0.15 (0.14)	0.05 (0.64)	0.13 (0.20)	0.13 (0.17)	0.06 (0.52)
Diastolic Blood Pressure	0.05 (0.64)	0.10 (0.30)	0.04 (0.71)	**0.23 (0.02)**	**0.24 (0.02)**	0.19 (0.05)
HDL-Cholesterol	0.076 (0.45)	0.001 (1.0)	−0.046 (0.65)	−0.149 (0.13)	−0.145 (0.14)	−0.180 (0.07)

Data are presented as R^2^ and (*p*-value)

*FBG* fasting blood glucose, *HbA1c* glycated hemoglobin, *HOMA-IR* insulin resistance, *HOMA-β* β-cell function

Table 3 shows the linear regression analysis of the impact of metabolic syndrome risk factors on diabetic parameters among FDRs of Type 2 DM. Both TG and WC significantly affected insulin levels, HOMA-IR, HOMA-β and HbA1c. In contrast, DBP had no effect on diabetic parameters.

**Table 3:** Impact of metabolic syndrome factors on diabetic parameters among FDRs of Type 2 DM

	HbA1c	Insulin	HOMA-IR	HOMA-β
Triglyceride	0.001 **(0.034)**	16.5 **(1.2 × 10**^**−6**^**)**	0.334 **(1.97 × 10**^**−6**^**)**	16.8 **(6.9 × 10**^**−5**^**)**
Waist Circumference	0.007 **(0.037)**	1.19 **(8.3 × 10**^**−6**^**)**	0.024 **(1.05 × 10**^**−5**^**)**	0.95 **(0.004)**
Diastolic Blood Pressure	−0.001 (0.814)	0.31 (0.284)	0.007 (0.273)	0.364 (0.322)

Analyzed by linear regression controlled for age and HDL-c, the results are presented as B value and (*p* value)

*HbA1c* glycated hemoglobin, *HOMA-IR* insulin resistance, *HOMA-β* β-cell function

## Discussion

The results presented in this study showed that TG and WC are the main metabolic syndrome factors that increased insulin resistance, β-cell function, insulin and HbA1c in non-diabetic FDRs of Type 2 DM. A recent study in healthy male adolescents with parental history of Type 2 DM demonstrated that elevated TG level and WC influenced the risk of insulin resistance [25]. Moreover, TG and WC were recently demonstrated to be associated with insulin resistance [26–29], with 75% of insulin resistance being attributed to the TG level and the association was significant even when TG was in the normal range, and 81.5 mg/dL was proposed to be the cut-off value of TG to predict the occurrence of insulin resistance [26]. Several early studies also showed insulin resistance to correlate well with TG levels [30, 31], and a dose-response relationship between serum TG and insulin resistance has been demonstrated [32]. Triglyceride was also reported to be an independent risk factor for insulin resistance and diabetes [27, 33] and an independent positive predictor of worsening insulin resistance and incident diabetes [34]. Elevated TG precedes impaired glucose regulation and that utilization of TG may identify individuals at high risk of incident diabetes earlier than standard glucose screening [35]. Similarly, WC was suggested to be used as a predictor of insulin resistance [28, 29, 36] with a cutoff value of 89.5 cm [37]; and that increased HOMA-IR was observed in subjects of normal BMI/high WC group [38]. Moreover, several studies showed that TyG index, a product of triglycerides and glucose, as well as TyG-related parameters that combined obesity indices and TyG index, such as TyG-BMI or TyG-WC, can predict insulin resistance [39–41] and be an early marker for detecting the risks of prediabetes and diabetes in FDRs of Type 2 DM [40].

Visceral (intra-abdominal) fat deposits are recognized to play a more important roles in the development of insulin resistance, because they produce more fatty acids and secrete inflammatory cytokines and adipokines [40, 42, 43]. Many studies have confirmed that higher levels of TG in the liver and muscle may disrupt glucose metabolism by increasing free fatty acids (FFA) and its flux from adipose to non-adipose tissue [44, 45]. It also limits insulin-stimulated glucose utilization by increasing fatty acid oxidation leading to a decreased synthesis of hepatic glycogen and reduced muscle glucose uptake [46]. Moreover, hypertriglyceridemia was reported to diminish glucose stimulated insulin secretion by inhibiting glucose oxidation via reducing pyruvate dehydrogenase (PDH) activity and elevating PDH kinase activity [47].

Several studies, in both humans and animal models, showed increased TG levels were associated with increased β-cell function [48, 49], and recent evidence showed TG to correlate with β-cell function in non-diabetic individuals [50] indicating the existence of severe insulin resistance and impaired β-cell compensatory response to insulin resistance. Moreover, in NGT subjects hypertriglyceridaemia was reported to be associated with increased insulin resistance and overstimulation of β-cell function [51]. Elevated TG levels in obese FDRs exhibited increased β-cell function, suggesting that serum TG is a good indicator of β-cell function in the newly characterized obese FDRs with NGT [52]. Earlier studies have also reported that impaired β-cell function caused by dyslipidemia precedes the manifestation of Type 2 DM [40, 53, 54].

Our results also revealed that non-diabetic FDRs of Type 2 DM patients to have insulin resistance and no signs of impaired β-cell function as reflected by the significantly higher HOMA-IR (87.5%) and HOMA-β (37.7%) with respect to the control subjects; suggesting that insulin resistance precedes the development of pancreatic β-cell dysfunction in individuals at risk of developing Type 2 DM. This is in agreement with several studies showing insulin resistance to precede the development of β-cell dysfunction [14, 15, 55, 56]. Moreover, results from a population-based prospective study in South Asian, showed that impaired glucose tolerance occurred 3–5 years after insulin sensitivity significantly decreased, whereas β-cell function remained unchanged during this period, signifying that insulin resistance may be the determinant factor in the progression of NGT to prediabetes [57]. A recent study in middle-aged and elderly Chinese population further supports that insulin resistance and β-cell dysfunction are the main determinants of developing prediabetes and Type 2 DM [58].

Insulin resistance preceding the development of β-cell dysfunction has been associated with more prevalent cardiometabolic disorders such as obesity, dyslipidemia, and hypertension [59] through mechanisms including low-grade inflammation, modifying lipoprotein particles, and impairing endothelial function [11]. Studies have also demonstrated that β-cell function could remain stable or exhibit high activity in obese individuals, mainly due to the β-cell compensation in response to insulin resistance [60, 61]. But the β-cell compensation may hit the limit with the continued deterioration of insulin resistance [62]. In contrast, several studies reported the degree of insulin resistance in FDRs to be similar to that of the newly diagnosed Type 2 DM, suggesting that progression from NGT to Type 2 DM in the non-obese FDRs was not attributed to the worsening of insulin resistance but to deterioration of β-cell function over time as the primary pathology [19, 21, 63–65]. This progressive deterioration in β-cell function was associated with a progressive increase in FFA and insulin resistance in adipose tissue [66].

Our results also showed non-diabetic FDRs of Type 2 DM patients to have significantly higher insulin levels with normoglycaemic levels, which are in agreement with earlier studies [9, 54, 67] showing ethnic groups with high susceptibility for Type 2 DM are prominently hyperinsulinemic with fasting glucose within the normal range [56, 68]. The observed plasma glucose, though within the normal range, were significantly higher in non-diabetic FDRs which is in support of earlier studies reporting FDRs of Type 2 DM to be at increased risk of developing hyperglycemia [14, 15, 69]. Thus as glucose tolerance gradually worsened in FDRs, the insulin levels and insulin resistance were also observed to rise progressively following the natural pathway of the disease [15, 70]. Moreover, Individuals with a family history of Type 2 DM showed higher plasma TG levels which are in agreement with several studies showing FDRs to have insulin resistance and dyslipidemia [15, 71, 72]. Higher prevalence of metabolic syndrome were also reported in FDRs as compared to those without a family history despite of normoglycemia [26, 73], and that hyperlipidaemic subjects were 3-times higher at risk of developing Type 2 DM [74].

Our results also showed plasma HbA_1C_ and fructosamine levels to be significantly higher in non-diabetic FDRs; which are in agreement with several studies [14, 15, 75]. The significant aspect of this increase was that it occurred within normal ranges of glucose concentrations. Our results also showed a significant correlation between HbA1c and HOMA-IR, which is in line with an earlier study [76] suggesting that HbA1c could be a marker not just for hyperglycaemia, but also for detection of insulin resistance. Different HbA1c cut-off values have been reported for diagnosing pre-diabetes and diabetes in different ethnic groups [77, 78]. Previous findings demonstrated that HbA_1C_ of 5.7 to 6.2 (upper tertile) was associated with reduced insulin action among non-diabetic, obese, FDRs of African-Americans who are genetically predisposed to Type 2 DM [79]. Moreover, HbA_1C_ of 5.45% was suggested to act as a predictive measure of impaired fasting glucose and metabolic syndrome as well as cardiovascular risk factors in the Korean population [76]. In addition, individuals with higher baseline HbA_1C_ values (≥5.8%) were reported to be at significantly higher risk for progression to impaired glucose tolerance and Type 2 DM [80–82].

Our study has some limitations. First, although the homeostasis model assessment of insulin resistance (HOMA-IR) has proved to be an essential tool for the surrogate assessment of insulin resistance, there is evident variability in the threshold levels of HOMA-IR to define insulin resistance. Second, HOMA-β is an indirect measure of β-cell function and only takes into account fasting/basal plasma glucose and insulin concentrations, thus generating limited information about the daily fluctuations in glucose homeostasis, and the impact of several common anti-diabetes treatments on either β-cell function or tissue insulin sensitivity. Third, all participants enrolled in this study were only male. Fourth, recall and misclassification biases may have affected our results, because the family history of diabetes data was obtained from participants by interviewers using questionnaires. Fifth, because our study sample consists mainly of Yemenis, the results cannot be generalized to other ethnicities.

## Conclusion

The results presented in this study showed that TG and WC are the main metabolic syndrome factors associated with insulin resistance, basal β-cell function, insulin and HbA1c in non-diabetic FDR men of Type 2 DM. Moreover, non-diabetic FDRs of Type 2 DM patients have insulin resistance with compensatory β-cell function (hyperinsulinaemia) suggesting that insulin resistance precedes the development of pancreatic β-cell dysfunction in individuals at risk of developing Type 2 DM. Our results also showed that metabolic syndrome may occur more frequently in non-diabetic FDRs and together with the existing insulin resistance and hyperinsulinaemia may participate in the development of β-cell dysfunction and the subsequent onset of diabetes.

## Data Availability

The data set generated and/or analyzed during this study are included in this submitted manuscript and is available from the corresponding author on reasonable request.

## Abbreviations

BMI: Body mass index
BP: Blood pressure
DBP: Diastolic blood pressure
ECl: Electrochemiluminescence immunoassay
FBG: Fasting blood glucose
FDRs: First degree relatives
FFA: Free fatty acid
HbA1c: Glycated haemoglobin
HDL-c: High-density lipoprotein cholesterol
HOMA-β: β-cell function
HOMA-IR: Insulin resistance
NGT: Normal glucose tolerance
SBP: Systolic blood pressure
SD: Standard deviation
SPSS: Statistical Package for Social Sciences
TG: Triglyceride
WC: Waist circumference
